# Movements of Blue Sharks (*Prionace glauca*) across Their Life History

**DOI:** 10.1371/journal.pone.0103538

**Published:** 2014-08-13

**Authors:** Frederic Vandeperre, Alexandre Aires-da-Silva, Jorge Fontes, Marco Santos, Ricardo Serrão Santos, Pedro Afonso

**Affiliations:** 1 Centre of IMAR of the University of the Azores; Department of Oceanography and Fisheries, Horta, Portugal; 2 LARSyS Associated Laboratory, Lisboa, Portugal; 3 Inter-American Tropical Tuna Commission, La Jolla, California, United States of America; Aristotle University of Thessaloniki, Greece

## Abstract

Spatial structuring and segregation by sex and size is considered to be an intrinsic attribute of shark populations. These spatial patterns remain poorly understood, particularly for oceanic species such as blue shark (*Prionace glauca*), despite its importance for the management and conservation of this highly migratory species. This study presents the results of a long-term electronic tagging experiment to investigate the migratory patterns of blue shark, to elucidate how these patterns change across its life history and to assess the existence of a nursery area in the central North Atlantic. Blue sharks belonging to different life stages (n = 34) were tracked for periods up to 952 days during which they moved extensively (up to an estimated 28.139 km), occupying large parts of the oceanic basin. Notwithstanding a large individual variability, there were pronounced differences in movements and space use across the species' life history. The study provides strong evidence for the existence of a discrete central North Atlantic nursery, where juveniles can reside for up to at least 2 years. In contrast with previously described nurseries of coastal and semi-pelagic sharks, this oceanic nursery is comparatively vast and open suggesting that shelter from predators is not its main function. Subsequently, male and female blue sharks spatially segregate. Females engage in seasonal latitudinal migrations until approaching maturity, when they undergo an ontogenic habitat shift towards tropical latitudes. In contrast, juvenile males generally expanded their range southward and apparently displayed a higher degree of behavioural polymorphism. These results provide important insights into the spatial ecology of pelagic sharks, with implications for the sustainable management of this heavily exploited shark, especially in the central North Atlantic where the presence of a nursery and the seasonal overlap and alternation of different life stages coincides with a high fishing mortality.

## Introduction

Sharks are generally characterised by a complex spatial organisation of their populations resulting from trade-offs between components of their life history, social and environmental interactions [1]–[5]. This complex organisation is reflected by their sexual segregation and the existence of discrete locations for key events during their life history, like pupping, nursing and mating. However, the existence and delineation of boundaries within shark populations and in particular the movements of individual sharks within and across these boundaries remain largely unknown. In the context of declining shark populations (e.g., [6]–[8]) and their deleterious ecological effects [9], unravelling such spatial organisation and, with it, accurately identifying Essential Fish Habitats, is key to develop appropriate management plans for the protection of the most vulnerable life stages [10]. This need is most compelling considering our current limitations in understanding the impacts of heterogeneously distributed fishing pressure on spatially structured shark populations [5], [11].

Blue shark is an oceanic predator with a global distribution [12] and a major constituent of the by-catch in pelagic longline fisheries [13]–[17]. The present status of blue shark stocks is the subject of much debate [18]–[21]. Blue sharks can be very abundant locally and have a productive life history strategy with a faster growth and a higher number of smaller offspring when compared to other pelagic sharks [21]–[24]. They are placental viviparous and females can give birth to up to 82 pups ([25]; mean litter size of 37 pups: [26]; up to 70 pups: [27]). Males and females mature at around 183 cm FL and 180–185 cm FL respectively [25], [26] at an age of 5–6 years [25]. However, sub-adult females (145 to185 cm FL) can engage in copulation and store spermatozoa in oviducal glands for later insemination, even if they still possess underdeveloped reproductive organs [25].

Results from conventional tagging studies have supported the conclusion, and concomitant management strategy, of a single blue shark stock in the North Atlantic (NA) [28], [29]. Yet, blue shark populations are known to segregate by sex and life stages and to make use of ecologically important areas [12]. Pupping areas are generally thought to be located off the western coasts of Portugal and North Africa [30], [31], although alternative locations near the Mid-Atlantic ridge in the central NA [17] and the Gulf of Guinea [26] have also been proposed. Although juveniles can be found in large parts of the NA, juvenile females appear to dominate in the eastern NA [32], [33] whereas juvenile males dominate in the western NA [18], [25], [34]. Mating has been recorded in the western NA [25], while observations of fresh bite marks indicating recent mating events [17], [25], [35] and the presence of dense male aggregations [36] suggests that mating also occurs in other areas.

These areas have all been identified based on analyses of fisheries dependent data and conventional tagging data. While these studies are fundamental in understanding the distribution across life stages, they tell us little about the dynamics and connectivity of these areas and are necessarily limited to those regions and seasons where fishing occurs. Furthermore, the definition of EFHs requires an evaluation of their ecological functionality. For example, nursery areas are generally considered to provide juveniles with an advantage, e.g. shelter or abundant food, thus increasing their possibilities to recruit to the adult population [37]. In practice, this is often difficult to evaluate [3], [37], particularly for pelagic sharks. Heupel et al. [3] therefore proposed a practical definition based on three criteria: (1) young-of-the-year sharks are more abundant than in other areas, (2) they have the tendency to remain or return for extended periods and (3) the area is repeatedly used across years.

Shark nurseries are typically closed bays or sheltered coastal areas that mainly provide protection from predators (e.g., [38]–[41]). In addition, nursery areas can be considered as one component of a shark's life history that, as a consequence of trade-offs between these components, would particularly benefit species with low breeding frequency, small size-at-birth, small litter sizes and/or low juvenile growth rates [3], [40]. It is therefore pertinent to test the practical definition proposed by Heupel et al. [3] on highly migratory, productive, oceanic species like blue shark [22]–[24].

Previous telemetry studies conducted on blue shark focussed on relatively short term (up to 210 days) horizontal and vertical movements [42]–[46]. In the Northwest NA, acoustic telemetry of mainly adult males showed consistent offshore and southerly movements between August and March and diel vertical niche expansion, extending hundreds of meters during daytime [42]. Juvenile and adult blue sharks of both sexes tagged in late summer and autumn off the eastern coast of Canada with PSAT tags were observed to expand their vertical niche as they entered the gulfstream and moved south and east to offshore overwintering grounds [46]. Juveniles of both sexes tagged in the English Channel and off southern Portugal during summer and autumn, displayed a general southward movement, with increased residence in frontal areas and behavioural plasticity in relation to their diel depth preference [44], [45]. Yet, because of the design and limited duration of the deployments, these studies provide us with little information about the seasonal movements and residency of different life stages and the complex spatial organisation of the NA blue shark population. In particular, longer-term data series are needed to validate the nursery assumptions.

Analyses of pelagic longline fishery data have indicated that the Azores region, in the central NA, is seasonally and alternately visited by immature, male and female adult blue shark, that it is important as a nursery ground and, potentially, also a pupping and mating ground for this species [17], [47]. The seasonal presence of all these life stages in a region with a central location in the NA offers an exceptional opportunity to investigate their movements, gain insights into the complex structuring of the NA blue shark population, and test the assumptions of the nursery concept in an oceanic shark.

This study addresses two objectives. The first was to investigate long-term migratory patterns of different life stages of blue shark, to study how their movements change across their life history, and to assess the connectivity between different areas they occupy. The second goal was to verify the existence of a nursery area for NA blue shark in the central NA, and to determine its boundaries and stability over time. A tagging experiment was set up in the Azores archipelago in which 37 blue sharks belonging to different life stages were tagged with satellite transmitters set for long-term deployments.

## Materials and Methods

### Ethics Statement

This study was performed according to national Portuguese laws for the use of vertebrates in research, and the work and tagging protocol approved by the Azorean Directorate of Sea Affairs of the Azores Autonomous region (SRAM 20.23.02/Of. 5322/2009), which oversees and issues permits for scientific activities. All procedures followed the guidelines for the use of fishes in research of the American Fisheries Society. The field studies did not involve endangered or protected species, no animals were sacrificed, and procedures for reduction, replacement and refinement were thoroughly adopted.

### 1. Tagging experiment

Blue sharks were captured onboard the R/V ‘Arquipelago’ (25 m) using a commercial style American longline (monofilament mainline and wire leaders) geared with 200 to 400 hooks, light sticks and baited with mackerel and squid. Fish selected for telemetry were then tagged off an auxiliary 7 m, low gunnel, fibreglass boat. Sharks were tagged at the surface after immobilization and induction of tonic immobility [48]. A total of 37 animals were tagged with different models of satellite tags, i.e. SPOT (Wildlife Computer SPOT5) and PSAT tags (Wildlife Computer MK10-PATs and Mini-PATs). SPOT tags were attached to the dorsal fin of male and female sharks measuring 127 to 211 cm FL through four nylon threaded rods fixed through stainless steel nuts and programmed to emit continuously on alternating days (one day on, one day off). MK10-PAT and Mini-PAT tags were attached intramuscularly under the first dorsal fin using a stainless steel tether and one of three types of anchors (small and large Wilton anchors, conventional titanium darts), and equipped with a guillotine to prevent descents bellow ca. 1800 m. MK10-PAT tags were deployed on males and females measuring 142 to 202 cm FL and programmed to release after 180 days. Mini-PATs were deployed on smaller individuals of 112 to 127 cm FL of both sexes and programmed to release after 180 or 270 days. Three individuals were double tagged with a SPOT and MK10-PAT tag.

The experimental design focussed on immature individuals in order to test the nursery hypothesis. However, it was also designed to address the movement patterns of different sexes and life stages of blue shark seasonally occurring in the area [17]. For this study, size limits of population segments, based on available knowledge of the species' biology [25] and seasonal occurrence [17], were as follows: (i) Small juveniles (SJ) up to 120–130 cm FL; (ii) Large juveniles (LJ) from 120 to 183 and 185 cm FL for males and females (i.e. including sub-adults females) respectively; and (iii) Adults (AD) above this latter thresholds. Four dedicated tagging cruises were performed during the four different seasons. Each cruise specific population segments were targeted for tagging, reflecting their seasonal availability (Table 1; Fig. S1: (i) SJ males and females (112–127 cm FL, n = 8) during summer and autumn; (ii) LJ females (127–178 cm FL, n = 9) during autumn and winter; (iii) LJ males (133–183 cm FL, n = 14) during summer and autumn; (iv) AD females (201–202 cm FL, n = 2) during winter and spring; and (v) AD males (200–211 cm FL, n = 4) during summer and autumn.

**Table 1:** Summary data for the 34 reporting blue sharks tagged off the Azores with satellite tags.

Shark	Deployment	Size (cm FL)	Sex	Life Stage	Class	Tag	Programmed Release (Days)	Recorded Release (Days)	Track Duration (Days)	Track Length (Km)	Argos Locations (n)	GLS Locations (n)	Data gaps (Days)	Recapture
1	17-10-2009	116	F	Juvenile	SJ	Mini-PAT	180	179	179	5316	0	54	74	
2	20-08-2010	122	F	Juvenile	SJ	Mini-PAT	270	102	102	3958	0	74	36	
3	20-08-2010	120	F	Juvenile	SJ	Mini-PAT	270	235	235	9346	0	208	17	
4	17-10-2009	116	M	Juvenile	SJ	Mini-PAT	180	180	180	5379	0	64	53	
5	20-08-2010	112	M	Juvenile	SJ	Mini-PAT	270	174	174	6295	0	124	36	
6	20-08-2010	127	M	Juvenile	SJ	Mini-PAT	270	193	193	6348	0	20	35	** 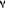 **
7	20-08-2010	120	M	Juvenile	SJ	Mini-PAT	270	273	273	8761	0	193	37	
8	21-08-2010	112	M	Juvenile	SJ	Mini-PAT	270	270	270	11533	0	220	38	
9	19-02-2009	127	F	Juvenile	LJ	SPOT			877	14494	236	0	389	
10	19-02-2009	139	F	Juvenile	LJ	SPOT			616	16907	779	0	21	
11	26-02-2009	175	F	Sub-adult	LJ	Double	180	91	226	8275	293	10	20	**^ 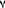 ^**
12	26-02-2009	142	F	Juvenile	LJ	Double	180	30	30	911	40	26	3	(** 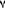 **)
13	26-02-2009	145	F	Juvenile	LJ	Double	180	89	952	28139	699	22	82	** 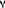 **
14	26-02-2009	148	F	Sub-adult	LJ	SPOT			90	2637	100	0	8	(** 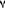 **)
15	06-03-2009	165	F	Sub-adult	LJ	MK10 PAT	180	3	3	76	0	2	3	** 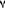 **
16	06-03-2009	156	F	Sub-adult	LJ	MK10 PAT	180	179	179	5307	0	41	31	
17	02-12-2009	178	F	Sub-adult	LJ	SPOT			579	15498	314	0	137	
18	16-10-2009	168	M	Juvenile	LJ	SPOT			36	1213	56	0	4	
19	16-10-2009	133	M	Juvenile	LJ	SPOT			206	5041	152	0	24	
20	16-10-2009	156	M	Juvenile	LJ	SPOT			82	2245	87	0	8	
21	02-12-2009	130	M	Juvenile	LJ	SPOT			42	973	41	0	6	
22	20-08-2010	164	M	Juvenile	LJ	SPOT			244	6892	196	0	22	
23	20-08-2010	180	M	Juvenile	LJ	SPOT			381	13066	387	0	16	
24	20-08-2010	140	M	Juvenile	LJ	SPOT			126	2364	189	0	6	** 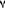 **
25	20-08-2010	183	M	Juvenile	LJ	SPOT			116	3846	160	0	9	
26	20-08-2010	183	M	Juvenile	LJ	SPOT			524	15066	612	0	32	** 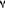 **
27	20-08-2010	159	M	Juvenile	LJ	SPOT			212	5994	332	0	10	
28	20-08-2010	172	M	Juvenile	LJ	SPOT			369	12451	610	0	12	** 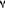 **
29	06-03-2009	202	F	Adult	AD	MK10 PAT	180	143	143	3577	0	2	89	
30	18-05-2012	201	F	Adult	AD	SPOT			33	1297	33	0	7	
31	16-10-2009	201	M	Adult	AD	SPOT			161	5108	133	0	22	
32	17-10-2009	200	M	Adult	AD	MK10 PAT	180	103	103	4338	0	100	13	** 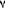 **
33	20-08-2010	207	M	Adult	AD	SPOT			228	6710	341	0	29	
34	20-08-2010	211	M	Adult	AD	SPOT			526	14500	929	0	24	

F – female; M – male; SJ – Small Juveniles; LJ – Large Juvenile, including sub-adults for females; AD – Adult; Double – Double tagged with SPOT and MK10 PAT tags; (**
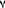
**) not confirmed recapture.

### 2. Analytical methods

Geographical positions of the SPOT tag transmissions and the popup locations of the PAT tags were obtained through the Argos system. Geographical positions from the PAT tags were reconstructed from archived light intensity curves transmitted by the tags after popup or retrieved after physical recovery of the tag. Geolocation (GLS, Global Location Sensing) was performed with the WC-GPE2 software, a program provided by the tag manufacturer (Wildlife Computers).

The raw tracks were post-processed using the IKNOS-WALK model [49]. In this approach, tracks are corrected and interpolated at fixed intervals through a non-state based random walk using a forward particle filter. In contrast with state-space models, no inferences are made about the unknown state of an animal in order to calculate subsequent positions [49]. Instead, locations are estimated from a cloud of weighted particles. The weighting can be manipulated to apply corrections based on known constraints (e.g. maximum speed) or available data. This flexible and intuitive approach has the additional advantage of being able to deal with both Argos and GLS positions and doing so taking into account the specific error distributions of each data type. In particular, longitude information is still preserved for position estimation during equinox, when latitude estimation is problematic for light based geolocation [50]. Another advantage over some methodologies is that, in case of data gaps, positions are interpolated in a straight line without over-fitting artefacts.

**Figure 1:**
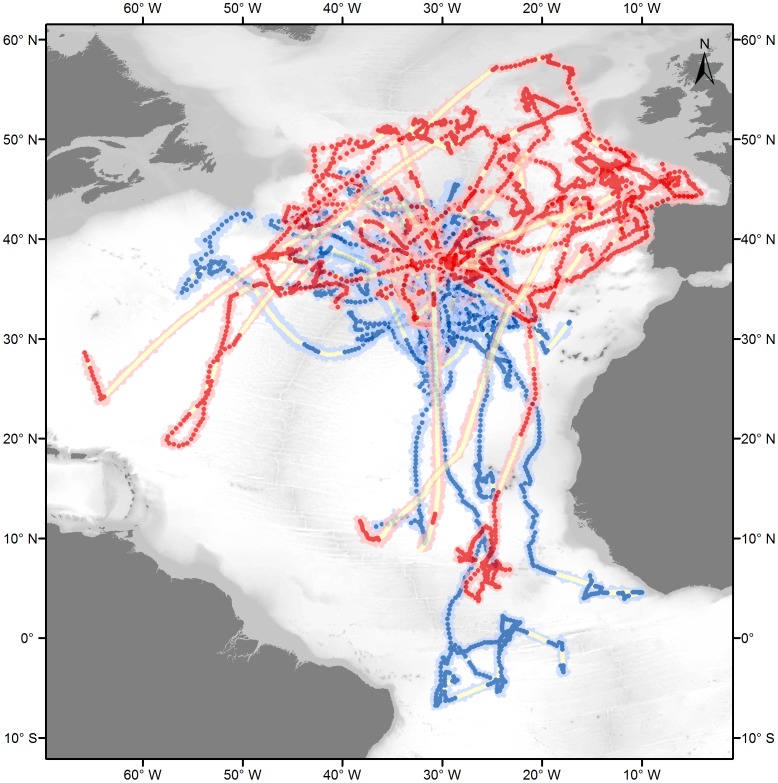
Reconstructed migratory pathways of blue sharks tagged in the Azores. Red and blue dots represent the most probable daily position estimates of respectively female and male sharks, with pink and light blue clouds representing the respective errors around the position estimates. Yellow dots indicate data gaps (i.e. days without position information within 72 h intervals).

The tracks were interpolated to obtain one daily position after imposing a maximum speed and impeding tracks from crossing land. The speed constraint is important for modelling the GLS positions, which have very large error distributions. It was set to 3 km/h based on speed estimates calculated from quality 2 and 3 Argos positions separated by time intervals of 1 to 4 days (Fig. S2) and movement information from the literature [42], [46], [51]. The error around estimated positions is represented by the dispersion of 50 time-matching alternative positions (represented as a cloud in Fig. 1). Fifty percent of the distances between modelled locations and ‘real’ GPS locations were reported to be less then 20 km and 104.8 km for Argos and GLS locations respectively [49]. The accuracy of the modelled tracks is further dependent on the quality of the track [49]. Consequently, an additional filter was applied on the modelled tracks in which interpolated points were considered to belong to a data gap when the dataset contained no raw location estimates within 72 hours from its time stamp (represented as yellow dots in Fig. 1).

In order to quantify and visualise the seasonal space use of the blue shark population segments, we calculated home range parameters for each of four quarters: from February to March, April to June, July to October and November to January. Fixed quarterly Kernel Utilisation Distributions (KUDs) were estimated for the different life stages based on aggregated locations of individuals within a given segment with deployments exceeding 90 days. KUDs were only calculated from estimated positions that did not belong to a data gap and for quarters for which more than 100 aggregated positions were available. All KUD calculations were performed using the adehabitatHR package [52] on the R statistical platform [53]. The remotely sensed SST data that we used in the analysis were obtained from NOAA's CoastWatch program and were provided as a monthly blended product from microwave and infrared sensors carried on multiple platforms (Japan's Advanced Microwave Scanning Radiometer AMSR-E, NOAA Advanced Very High Resolution Radiometer AVHRR, the Imager on NOAA's GOES spacecrafts, and the Moderate Resolution Imaging Spectrometer MODIS aqua) with complete coverage and a spatial resolution of 0.1 degrees. Subsequently, the SST data were averaged to a resolution of 1° using the arithmetic mean.

## Results

### 1. Tag performance

In total we tagged 37 blue sharks with 40 tags, of which only three MK10-PAT tags deployed on LJ males failed to report (Table 1). SPOT tags (n = 21) reported for periods between 31 and 952 days (median = 220 days), eight of which for over one year (>365 days), resulting in a cumulative transmission period of 6429 days. While these SPOT transmission periods are long, some tracks showed considerable data gaps. The reporting MK10-PAT tags (n = 7) released after 3 to 180 days (median = 91 days). The number of light intensity curves received through the Argos system and the actual number of GLS positions that WC-GPE2 was able to calculate varied greatly between tags (2–41 GLS locations). Access to the full archive of one recaptured tag (Shark 32) allowed for the calculation of 100 GLS positions. Mini-PATs generally performed better than the MK10-PAT tags, resulting in longer deployments of 102 to 273 days (mean = 201 days). Transmission and data quality were also higher than for the MK10-PAT tags resulting in 20 to 220 GLS locations per tag.

### 2. Recaptures

At least 8 sharks (21.6%) were recaptured by commercial fishermen anywhere from 3 to 952 days after tagging (Table 1). Recaptures were either reported by the fishermen or confirmed by the detection of Argos tracks typical of fishing operations by commercial longliners. An additional two sharks were possibly recaptured, but this could not be verified.

### 3. Movements

**Figure 2:**
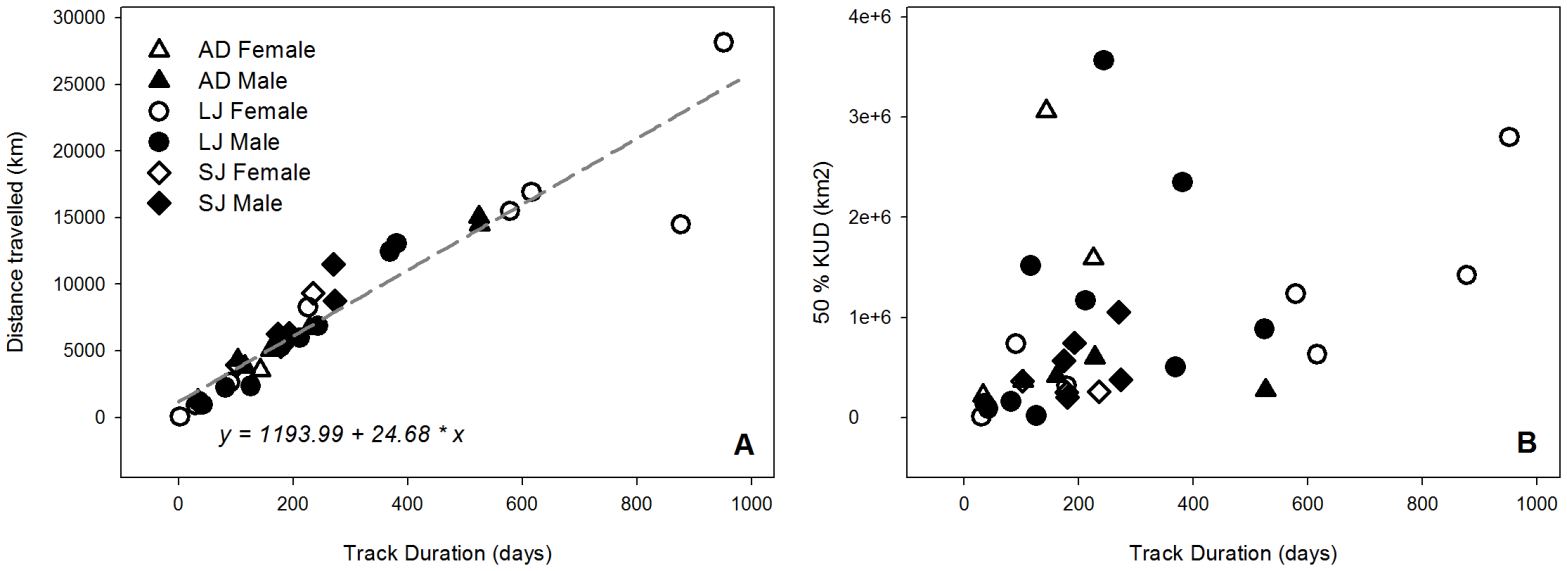
Distance travelled (A) and 50% fixed Kernel Utilisation Distributions (KUD) (B) in function of the track duration for the blue sharks belonging to different life stages. AD – Adults; LJ – Large juvenile; SJ – Small juvenile.

In general, blue sharks tagged in this study displayed wide ranging movements (Fig. 1). The longest deployment was registered for a LJ female (Shark 13, 145 cm FL) that travelled an estimated 28139 km over a period of 952 days. All sharks were oceanic, with individuals exploring shelf break areas, but none apparently moving onto continental shelves. While the tracks revealed important individual variability (Fig. 1), the distance travelled as a function of time at liberty was not significantly different between individuals or life stages (Fig. 2A). The only outlier was shark 9, of which we lost track during 389 days.

#### 3.1 Small Juveniles (SJ)

**Figure 3:**
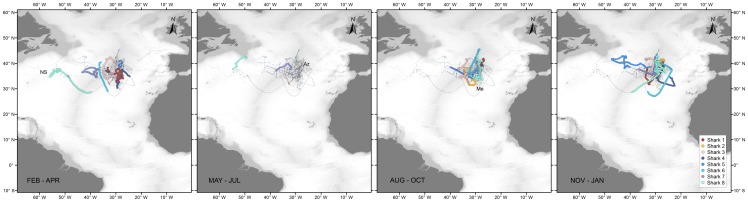
Reconstructed movements of small juvenile male (green – blue) and female (yellow – red) blue shark tagged in the Azores by quarter. Grey lines represent track segments of the individuals during other quarters. Az – Azores, Me – Great Meteor Seamount; NS – New England Seamounts.

SJ sharks of both sexes were tagged with Mini-PAT tags providing low resolution location data (GLS). In general, no differences are apparent between the quarterly movements of SJ males and females (Fig. 3). Both sexes used a relatively limited area, comprised between 25° and 45°N and 22° and 56°W for up to 235 days. Two sharks (sharks 1 and 4), a male and a female tracked for a period of 6 months between October 2009 and May 2010 remained mainly in an area south-southwest of the Azores, near the MIDAR and the Atlantis – Great Meteor seamount complex. The six sharks, four males and two females tagged in August 2010 and tracked for periods between 101 and 270 days, spent most time in this same general area. Two males (sharks 7 and 8) initiated a westward movement during January. Shark 8 moved as far west as the eastern edges of the New England seamounts, before we lost its track near the southern slopes of the Grand Banks at the end of May. Shark 7 moved to an area close to 36.5°N 43°W, before returning to the same general area south-southeast of the Azores in May. Shark 5 moved to the Newfoundland Basin during autumn and returned by January.

#### 3.2 Large Juveniles (LJ)

**Figure 4:**
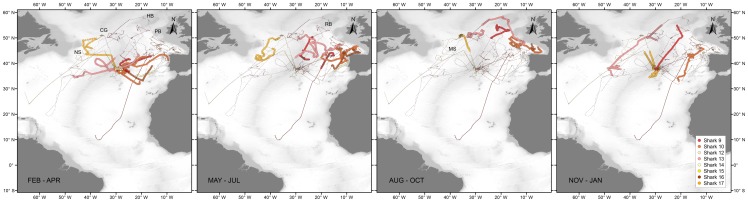
Reconstructed movements of large juvenile and sub-adult (LJ) female blue shark tagged in the Azores by quarter. Grey lines represent track segments of the individuals during other quarters. NS – Newfoundland Seamount; CG – Charlie-Gibbs Fracture Zone; HB – Hatton Bank; PB – Porcupine Bank; RB – Rockall Bank; MS – Milne Seamount.

**Figure 5:**
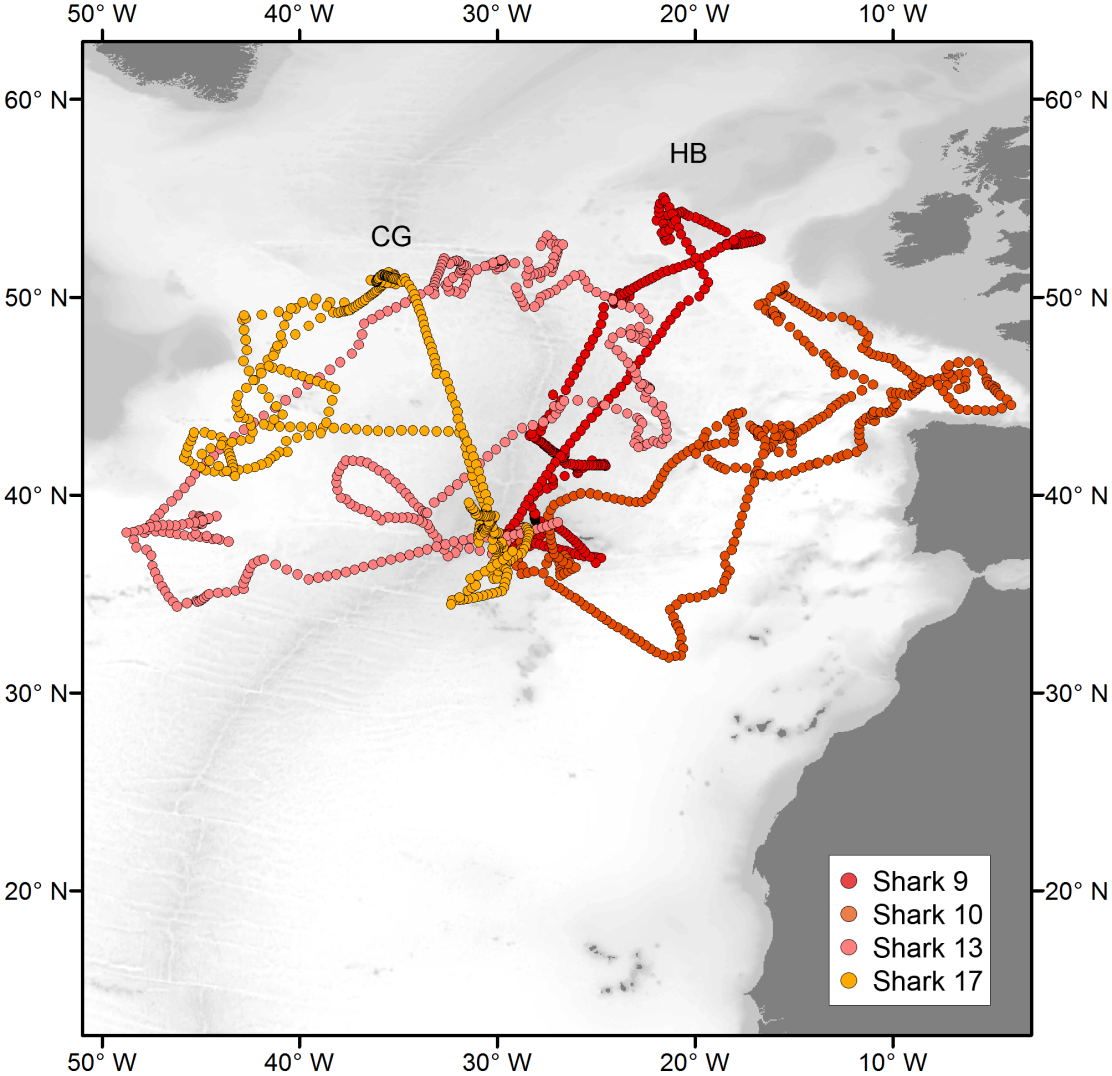
One year reconstructed tracks of four large juvenile and sub-adult (LJ) female blue shark tagged in the Azores. CG – Charlie-Gibbs Fracture Zone; HB – Hatton Bank.

Large juvenile females were tagged in autumn (2009, n = 1) and winter (2009, n = 8) with SPOT and/or MK10-PAT tags, providing high resolution location data (Argos data) for most individuals (sharks 9–15 and 17). In contrast with the SJ of both sexes, LJ females performed extensive latitudinal movements, utilising an area comprised between 31°N and 59°N and 3°W and 50°W (Fig. 4). LJ females displayed marked seasonal migrations, residing in the southern part of their range during winter (i.e. between 31°N and 45°N) and moving northward during summer (i.e. between 45°N and 59°N). During the latter season, LJ females explored mainly seamount and slope areas in the north-eastern NA, from the Bay of Biscay to the Hatton bank (sharks 9, 10, 13 and 16), near the Charlie-Gibbs Fracture Zone (sharks 13 and 17) and east and south of the Flemish cap (sharks 13 and 17). Four individuals (three juveniles and one sub-adult, sharks 9, 10, 13, 17), were tracked for over a year allowing the observation of a full seasonal cycle (Fig. 5). All four sharks returned to the Azores approximately one year after release, despite having moved to different areas during summer. Whereas shark 10 moved to the eastern NA during two consecutive summers, in the same period shark 13 explored almost the entire longitudinal range between the Flemish cap and the Bay of Biscay. Sharks 12 and 15 were probably both recaptured close to the tagging site 30 and 3 days after release, respectively. Shark 14 moved to the Iberian coast.

**Figure 6:**
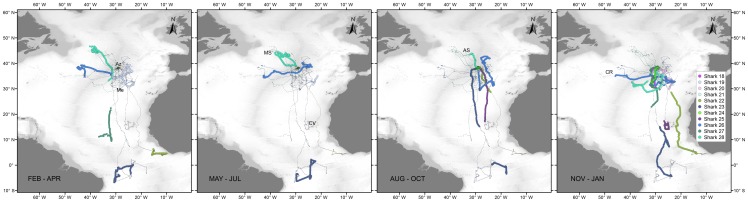
Reconstructed movements of large juvenile (LJ) male blue shark tagged in the Azores by quarter. Grey lines represent track segments of the individuals during other quarters. Az – Azores; Me – Great Meteor Seamount; MS – Milne Seamount; AS – Altair Seamount; CR – Corner Rise Seamounts.

LJ males were tagged with SPOT tags during summer (2010, n = 7) and autumn (2009, n = 4), providing high resolution location data for all sharks. This population segment also displayed extensive movements, but the seasonal nature of these movements appeared less pronounced as for LJ females (Fig. 6). Nonetheless, by December, 10 of 11 LJ males started moving south after spending up to 105 days (median = 37 days) in an area close to the tagging location. The core of this area, located south-southwest of the islands of Faial and Pico, is constituted by a complex of seamounts and offshore banks (e.g. Condor Seamount, Açores Bank and Princess Alice Bank) that joins the MIDAR further to the southwest. We will refer to this core area as the Princess Alice complex. Four sharks (sharks 21, 18, 20 and 24) were lost between 21 and 46 days after they initiated their southward movement. At that time these fish were located approximately at the latitude of Great Meteor Seamount, 8 degrees south of the tagging location. After the initial southward movement, four of the remaining individuals (sharks 22, 23, 25 and 27), dispersed further south to tropical latitudes with one shark eventually moving to the South Atlantic, where it remained 8 months. In contrast, two sharks that were tracked for 524 and 369 days (sharks 26 and 28, respectively), remained in the area south-southwest of the Azores after their initial southward movement in autumn. At the end of January, both sharks initiated a westward movement to areas in the north-western NA, where they remained for approximately 1.5 months before returning towards the Azores during May. The following autumn, shark 26 moved again to the area south-southwest of the Azores and initiated a new westward movement in December towards the Corner Rise Seamounts. In contrast with the other LJ males, shark 19 (140 cm FL) exhibited movements similar to SJ sharks, remaining in a smaller area close to the Archipelago during the entire duration of the deployment (206 days).

#### 3.3 Adults

**Figure 7:**
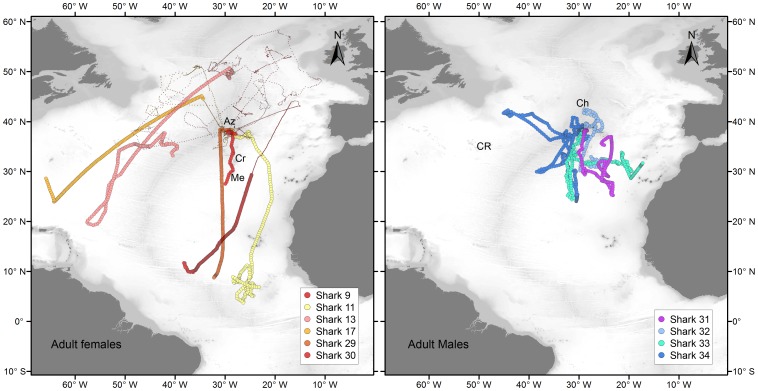
Reconstructed movements of adult (AD) male (right) and female (left) blue shark tagged in the Azores by quarter. Grey lines represent track segments of the individuals during other quarters. Az – Azores; Me – Great Meteor Seamount; Ch – Chaucer Seamount; Cr – Cruiser Seamount; CR – Corner Rise Seamounts.

Two AD females (shark 29, 202 cm FL; shark 30, 201 cm FL) and one large LJ female (shark 11, 175 cm FL) were tagged in winter (2009, n = 2) and spring (2012, n = 1) with SPOT and/or MK10-PAT tags, providing high resolution location data for two individuals (sharks 11 and 30). Shark 30 released fully developed pups during capture, indicating late stage pregnancy at the time of tagging. All three individuals migrated south within 33 days after tagging (Fig. 7). After remaining 135 days (June to October) in tropical waters, in an area comprised between 3.5°N–11°N and 23°W–28.5°W, shark 11 was recaptured by a fisherman who reported that the shark was carrying embryos at the time. Similar southward migrations towards tropical and sub-tropical waters were also observed for 3 juvenile and sub-adult females that were tracked for over a year. After 830 days of tracking, shark 9 reappeared for a period of 29 days in tropical waters near 10°N 36°W in June (2011) after its signal had been lost for 389 days. Sharks 13 and 17 both migrated to the southern part of the Sargasso Sea by June 2011 after approximately 826 and 526 days, respectively. Shark 13 moved back north towards the Corner Rise Seamounts and then east towards the MIDAR near 33°N and 40.5°W, where we lost its track in October 2011. The southward migrations of sharks 9, 13, 17 and 29 were accompanied by large periods (137, 82, 389 and 89 days respectively) when no Argos locations or good GLS locations were obtained and reconstructed migratory pathways were difficult to produce.

The movements of the AD males were similar to those observed for LJ males (Fig. 7). Four adult males were tagged during summer (2010, n = 2) and autumn (2011, n = 2) with SPOT or MK10-PAT tags, providing high resolution location data for three sharks (sharks 31, 33 and 34). By December all four individuals started moving south. Before that time, three sharks (sharks 31, 33, 34) spent between 14 and 117 days within the Princes Alice complex. Shark 32 also remained in the vicinity of the archipelago. Thereafter, all four sharks stayed in the area south-southwest of the Azores, mainly near the MIDAR and the Atlantis – Great Meteor seamount complex, but moving as far south as 24°N, at least until the end of January. Shark 31 returned to the Archipelago by the middle of March, when we lost its signal. Shark 33 moved east until we lost its track between Madeira and the Canary Islands at the beginning of April. Shark 34 moved west towards the north-western NA during the month of February, reaching an area south of the Newfoundland Seamount in March. The second summer the shark moved again to the Azores, spending most time within the Princes Alice complex, initiating a new southward movement during November.

### 4. Space use

**Figure 8:**
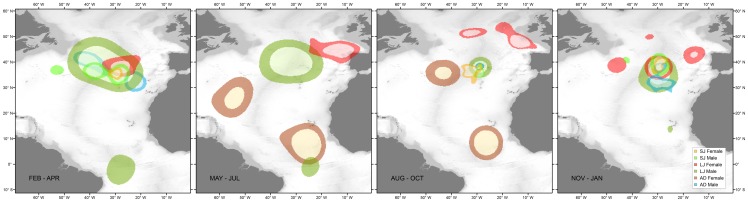
Quarterly 25% and 50% Kernel Utilisation Distributions (KUD) for the different life stages of blue sharks tagged in the Azores. Orange – Small Juvenile (SJ) females; Green – SJ males; Red – Large Juvenile and Sub-adult females; Dark green - Large Juvenile (LJ) males; Brown – Adult (AD) females; Blue – AD males.

**Figure 9:**
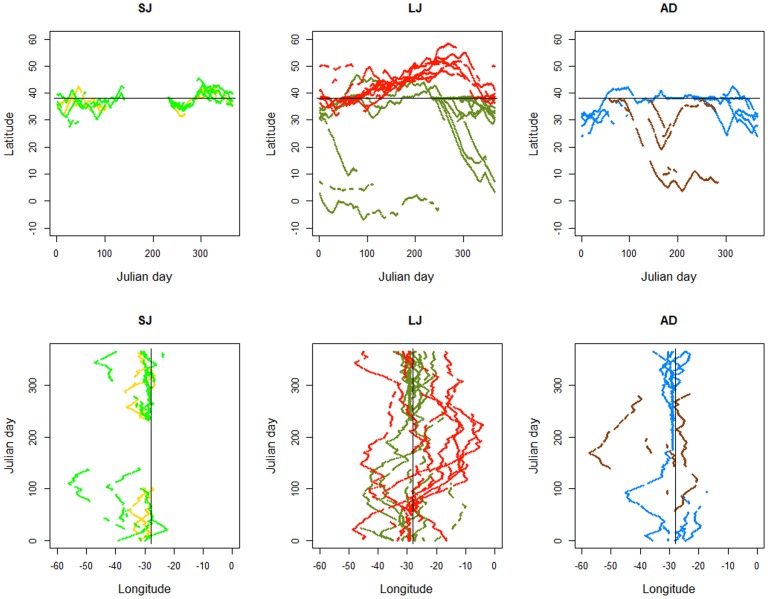
Latitudinal and longitudinal displacements by day of the year of different life stages of blue sharks tagged in the Azores. Orange – Small Juvenile (SJ) females; Green – SJ males; Red – Large Juvenile and Sub-adult females; Dark green - Large Juvenile (LJ) males; Brown – Adult (AD) females; Blue – AD males.

Distance travelled as a function of time at liberty was similar across individuals (Fig. 2A) but the concomitant variation on home range size changed dramatically (Fig. 2B). The 25% and 50% KUD areas were highly variable both between sexes and life stages (Fig. 8). The latitudinal and longitudinal displacements over time (Fig. 9) further illustrate the contrast in spatio-temporal dynamics across the species' life history. SJ of both sexes were contained within a narrow latitudinal range and mainly utilised an area delimited by the Azores archipelago to the North, the Atlantis – Great Meteor seamount complex to the South, and the MIDAR to the South-West. There was little difference between sexes, except that males extended their range more towards the West. In contrast, LJ of both sexes showed a wider range in both latitude and longitude. The segregation between both sexes becomes apparent during summer and autumn, when females extended their range to the North and East and males to the South. Conversely, AD females moved South and South-West while AD males used a more limited range, mainly extending their distribution South and West of the tagging location. The figures also clearly illustrate the intermediate geographical position of the tagging location as the distributions of all life stages spatially overlap in this area, albeit in different quarters.

### 5. Time at SST

**Figure 10:**
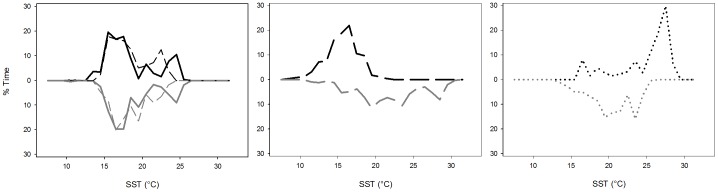
Aggregated long-term time at Sea Surface Temperature (SST, °C) profiles for male (grey) and female (black) blue sharks belonging to different life stages. Top panel: Small Juveniles (SJ); Centre panel: Large Juveniles and Sub-adults (LJ); Bottom panel: Adults (AD). SST values were obtained from averaged (1°) monthly SST images for NOAA's CoastWatch program, except for the dotted curves in the top panel that were obtained directly from the Mini-PAT tags.

The spatio-temporal segregation patterns are also reflected in the pooled time-at-SST profiles for the different population components (Fig. 10). No differences were apparent between SJ males and females, which show two preferred maxima, i.e. approximately 17°C and 25°C, corresponding to the most common temperatures within the narrow latitudinal range they occupied. The figure also shows a discrepancy between the SST recorded by the Mini-PATs and that obtained from remote sensing, which is probably due to heating of the surface layer as a consequence of increased sun exposure and low wind speeds during summer. In contrast, the temperature niche segregation between male and female LJ was evident, with males seemingly preferring warmer waters between 16°C and 30°C. This SST range was roughly identical as for AD males, whereas AD females used a wider temperature range (15°C to 30°C) and, apparently, preferred warmers waters within it (25°C to 30°C). However, this pattern is based on a smaller dataset and seasonal coverage.

## Discussion

The blue sharks tagged in the Azores displayed extensive movements over vast areas of the North Atlantic, with one individual eventually moving into the southern hemisphere. The sharks appeared to be mainly oceanic and to explore shelf break areas, but did not venture onto the continental shelves, although many studies about blue shark catch data and conventional tagging are from shelf areas (e.g., [18], [28], [30], [34], [54]). This finding supports the conclusions of previous studies that pointed out the potential bias that release sites from conventional tagging studies can introduce in the interpretation of movements [12]. Furthermore, the sharks in this study exhibited considerable individual variability in their movements, both among individuals and life stages as well as individually across time.

### 1. Testing the nursery concept

Our data showed that both male and female SJ blue shark tend to remain for extended periods of time (up to 235 days) in a general area delimited by the Azores, the Atlantis – Great Meteor seamount complex and the Mid-Atlantic Ridge (MIDAR). Additionally, the movement patterns and SST preference did not show any indications of segregation between the two sexes at the spatial scale considered in this study. This pattern is in agreement with available data from fisheries-dependent conventional tagging programs, which suggest that juveniles of 100–130 cm FL do not participate in extensive latitudinal migrations [12], [28], [30], [32], [55]. Nonetheless, Queiroz et al. [44] found movements of juvenile blue shark closer to continental shelves to be more extensive, and Litvinov [36] concluded that male and female blue shark segregate during their first year, at lengths smaller than 70 cm FL. This is in disagreement with the results from the present study and a recent demographic analysis of fisheries data from the Azores region [17]. Both studies indicate that segregation does not occur before, at least, the second year, when blue sharks start to take part in larger scale migrations.

Our study does not present indications of connectivity between the central NA and other juvenile grounds, such as the continental shelves of the Iberian Peninsula and Northern Africa [12], [32], reinforcing the hypothesis that parturition may also take place in this area of the NA [17]. Moreover, our study provides additional evidence of the existence of a nursery area for blue shark in the central NA. Not only are juvenile blue sharks locally abundant across years [17], [47], but individual blue shark have the tendency to remain and return to the area for extended periods, meeting the three criteria proposed by Heupel et al. [3]. Still, in light of the classical notion of shark nursery areas [40], the classification of such a vast offshore area as a nursery could be disputed. Even so, oceanic nurseries have previously been suggested for some oceanic species, but solely based on observations of small individuals and/or pregnant females in offshore areas, e.g. salmon shark, *Lamna ditropis* [56], [57], bigeye thresher, *Alopias superciliosus* [58], and oceanic whitetip, *Carcharhinus longimanus* [59]. Despite the consistent spatial patterns observed, our study was not designed to clarify whether the area provides juveniles with increased survival probability by providing them shelter from predators or some other advantages [3], [37]. Oceanic sharks have typically larger litters of smaller young than their coastal counterparts, which has been interpreted as an adaptive strategy of the offspring as a whole to thrive on patchy oceanic resources, benefiting from the supposedly lower predation pressure in the open ocean [60]. Lower predation risk in oceanic waters is also seen as an evolutionary driver for the oceanic juvenile stage in some sea turtles [61], [62]. A favourable habitat providing increased growth rates and thus a reduced time at vulnerable sizes [3] therefore appears to be a plausible advantage for an oceanic nursery habitat of blue shark.

Juvenile survival has been shown to have great impact on blue shark population growth [24]. Due to their small size-at-birth, juvenile blue sharks are probably highly vulnerable during their first year of life [22], which could be balanced through rapid juvenile growth [24], [40]. While food is generally not considered a limiting factor for juvenile sharks in typically prey rich estuarine and coastal nursery areas [40], it has been shown to limit the survival of juvenile scalloped hammerheads in a Hawaian nursery [41] and may play an important role in oceanic and oligotrophic environments. It can therefore be argued that the area provides juvenile blue shark with optimal growth conditions supported by ample food resources associated with the diversity of topographic features (seamounts and islands) [63] and localised oceanographic processes [64]. In addition, the intermediate geographical location of the wider Azores ensures a favourable temperature niche throughout the year. This combination of factors is probably what confers the region an important role for other highly migratory species, e.g. blue and fin whales [65] and juvenile loggerhead sea turtles [66], [67]. Remarkably, the distance travelled by these SJ sharks as a function of time is similar to that of other life stages. This finding strongly suggests that their limited distribution when compared to that of larger life stages is unlikely to be merely a consequence of limited swimming capacities. In conclusion, our study provides strong support for the existence of an oceanic shark nursery, although further research is needed to clarify its primary drivers. The designation of the wider Azores as a nursery area also seems meaningful from a management perspective, considering the spatial scale at which the pelagic longline fishery operates.

### 2. Movements into adulthood

LJ females are known to undertake large scale latitudinal migrations, moving to northern latitudes during summer [12], [30], [32], [33]. In the NA, they dominate the summer catches off south-west England and are present off the US and Canada [18], [25], whereas in winter high abundances are mainly recorded off Portugal [55] and around the Azores [17], [47]. This putative migratory pattern was directly observed in all of our tagged LJ females with periods at liberty exceeding 90 days (n = 6). Although the summer patterns revealed considerable individual variability as individuals explored almost the entire longitudinal range across the NA from the Flemish Cap to the Gulf of Biscay, LJ females displayed a strong connectivity with the eastern NA as indicated by conventional tagging [54]. Regardless of this variability, the four sharks that retained the transmitters for at least one full year were all tracked back to the Azores region during winter, indicating a high degree of site fidelity to the region. LJ females stayed within colder temperatures than younger or older sharks (10°–20°C) and showed a well marked preference for surface waters with an SST between 15° and 16°C throughout the year. The affinity of blue shark for certain water temperatures has been extensively described in the literature although these studies rarely made the same distinction between life stages as we do, making it difficult to compare findings. Nonetheless, highest abundances of juvenile and sub-adult females in the Northwest and central NA were recorded within the same range [17], [18].

Tagging AD female blue shark was not the prime objective of the present study. Nonetheless, the few large sub-adult and adult female that we tagged all displayed directed southern movements to tropical latitudes in sharp contrast with LJ females. One AD female was later recaptured in tropical waters carrying embryos, indicating that these movements are related to the reproductive cycle. Additionally, we were able to observe a behavioural switch in three females up to 2.5 years after release, when they reached the theoretical adult size between 170 and 200 cm FL [68]. Up to that point, these sharks had remained in cold waters migrating between sub-tropical and temperate regions, after which all of them moved to southern tropical grounds in June and July utilising pathways on both sides of the ocean. Female blue sharks are thought to move offshore from coastal areas in the north-western NA after mating and to migrate towards the eastern NA to mature and deliver pups [18], [25]. However, our results also show that a proportion of mature females move South towards the south-western NA, where their arrival in early summer coincides with the increasing seasonal catch rates of mature and pregnant females [27]. This finding supports the hypothesis of a southbound movement of mature blue sharks into the south-western NA as inferred from conventional tagging data [12], [29]. In the eastern NA, adult and pregnant females are seasonally observed in different stages of the reproductive cycle. Mature and pregnant females are found during winter near the Canary Islands and African coast, with mating and pupping believed to occur off Portugal [12] and near the Azores during spring [17]. Castro and Mejuto [26] found a large proportion of adult females in the Gulf of Guinea, many of which were pregnant. The present study indicates that small adult females also move to offshore areas further south (3°–12°N) into the south-eastern NA. This is in agreement with their presence in tropical latitudes [26], [27], a behaviour that was hypothesised to facilitate fertilization and embryonic development [69].

LJ and AD males displayed similar movements and do not appear to segregate spatially. After spending up to 117 days within the Princes Alice seamount complex, both groups started moving south to an area south-southwest of the Azores during autumn. This autumn migration coincides with a decrease in abundance of both life stages in local catches [17]. Accordingly, Litvinov [70] observed high abundance of adult males above the seamounts south of the Azores during autumn and labelled these concentrations as “male clubs”. In fact, three sharks (2 LJ and 1 AD) remained in that area until January before initiating a westward movement to offshore areas in the western Atlantic. By the end of spring these fish returned to the Azores, eventually resuming a new southward movement at the end of summer. Such a clear cyclic pattern of migration appears to be common throughout the life history of male blue sharks, as it was also observed in small juveniles. It demonstrates the strong connectivity between the north-western and central NA for males, as previously hypothesised [28], [71].

In the northwest Atlantic, adult and sub-adult blue shark enter coastal waters during late spring and summer and engage in mating [25]. Thus, the subsequent migration of males from the central NA to waters off the north-western NA in late spring could well be related to such mating. The behavioural shift in spring of two tagged females in these off-shore areas appears to support this hypothesis, but their capacity to store sperm would not require such a temporal matching of events. In addition to the western migration cycle, LJ males also exhibited an important southward dispersal, which is in accordance with their dominance in tropical waters [26], [27].

### 3. Underlying mechanisms of blue shark spatial ecology

The long-term tracking of blue shark in this study revealed the existence of a high degree of site fidelity with the central NA across almost all life stages, except for adult females for which no long-term tracks were available. Such site fidelity appears to be common in migratory shark species (e.g. great white shark, *Carcharodon carcharias* [72], tiger shark, *Galeocerdo cuvier* [48], broadnose sevengill shark, *Notorynchus cepedianus* [73]) and other pelagic predators like tuna [74] and cetaceans [75] and is generally associated with feeding, parturition or mating. The identification of these specific areas is considered an important step for the management of highly mobile species [76] and the fact that site fidelity towards the central NA is present throughout its life history emphasises the importance of the area for blue shark.

The cyclic migrations and dispersal to tropical latitudes of LJ and AD males suggest some degree of behavioural polymorphism within the male population supported by what seems to be a high tolerance and adaptation to habitat conditions. In fact, the wide geographical range occupied by these males was also reflected in their broad SST niche (12–30°C and 15–30°C, respectively), in agreement with fisheries catch analysis [17]. Such behavioural polymorphism, also observed in many fish (e.g., [77]) and sharks [48], may have ecological reasons, such as the existence of different feeding or reproductive strategies [48]. Given the segregation between AD and LJ females, one hypothesis to explain the behavioural polymorphism in male blue shark could therefore be the co-existence of different reproductive strategies aimed at mating with either of the two female life stages. Indeed, AD females are seldom observed at the mating areas in the north-western NA, where LJ females occur (see above) which are visited by males exhibiting the western migration cycle. Conversely, LJ females are virtually absent in tropical latitudes to where both AD females and (some) males appear to converge. This possibility casts a previously unknown complexity on the behavioural ecology of pelagic sharks and blue shark in particular.

The patterns of spatial segregation between sexes, which are considered a common trait in sharks [1], [2], [38], [39], change over the course of the blue shark's life cycle indicating that the relative importance of the drivers behind the segregation changes along with it. This is particularly so for females, which undergo pronounced ontogenetic shifts in distribution and movement patterns during their juvenile phase as well as upon reaching maturity. Contrastingly, male blue sharks expand their range, mainly to warmer waters during their juvenile phase, but typical movement patterns are observed throughout their life history. As a result, LJ females segregate more clearly from other life stages, occupying colder waters and mainly occurring together with smaller sized individuals of both sexes [17], [55]. Because this is a juvenile life stage and growth is similar for males and females, this pattern is likely a means for LJ females to avoid aggressive courtship behaviour until approaching sexual maturity [2], [5], which is also why females develop an epidermis twice as thick as that of males [25]. The behavioural shift observed in females upon reaching maturity could then be related to the search of warmer thermal habitat to aid fertilisation and embryonic developments as proposed by Hazin et al. [69]. Other drivers in shaping the segregation patterns in blue shark can not be excluded (see [2], [5] for reviews), and it is possible that certain patterns were not observed because they act at different spatial scales than could be detected in the present study.

## Conclusion

The long term tracking of different life stages performed in this study demonstrates the complex structuring of the NA blue shark population. The study builds upon previous studies [17], [47], providing strong evidence for the existence of a discrete central NA nursery roughly delimited by the Azores archipelago in the North, the Atlantis –Great Meteor seamount complex in the South and the MIDAR in the South-West. This oceanic nursery is used by juveniles of both sexes, at least during their first year, after which they start to show the typical segregation pattern that can be seen in the older juvenile component. Upon reaching maturity, females move to southern latitudes utilising migration pathways on both sides of the NA. Males extended their range, displaying a high connectivity with the western NA and dispersal to tropical latitudes. Moreover, there is a high degree of site fidelity with individuals of nearly all life stages returning to the central NA, emphasizing the importance of this region and their possible role as pupping grounds. Such complex spatial structuring and philopatric behaviour will have important consequences for the management and conservation of blue shark [11], [78] in the Atlantic, especially considering that the seasonal overlap and alternation of different life stages in the central NA nursery coincides with high levels of fishing mortality [79].

## Supporting Information

Figure S1 **Overview of the tagging experiment.**
(TIF)

Figure S2 **Frequency distribution of blue shark speeds (km/h).** Speed (km/h) was calculated as the displacement between quality 2 and 3 Argos locations separated by time intervals of 1–4 days.
(TIF)
