# Supplementary figures and images for: Movements of Blue Sharks (Prionace glauca) across Their Life History
Source: PLoS One. 2014 Aug 13;9(8):e103538. doi: 10.1371/journal.pone.0103538 (PMC4131881; doi:10.1371/journal.pone.0103538)

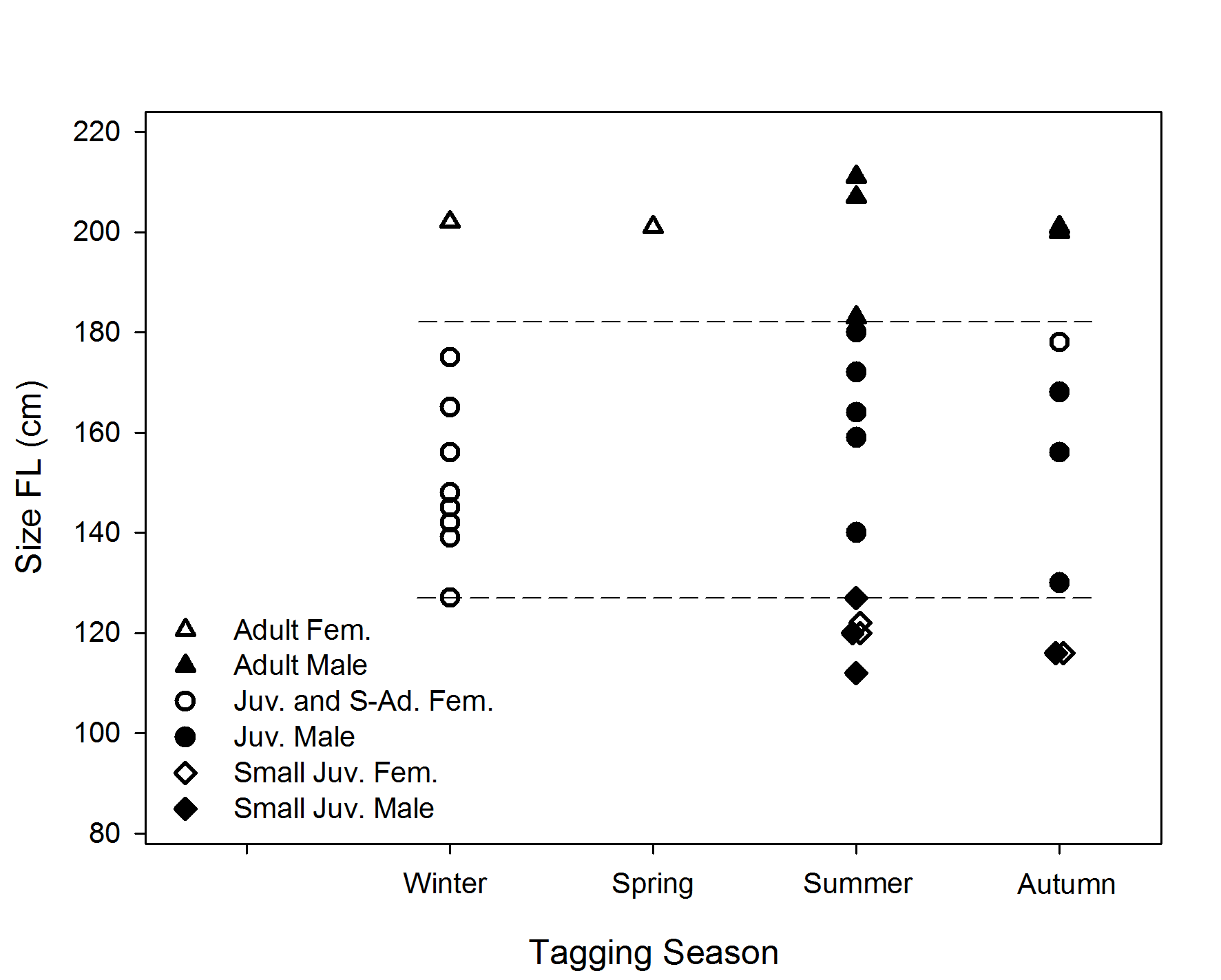

Supplement: Figure S1 — Overview of the tagging experiment. (TIF) [file pone.0103538.s001.tif]

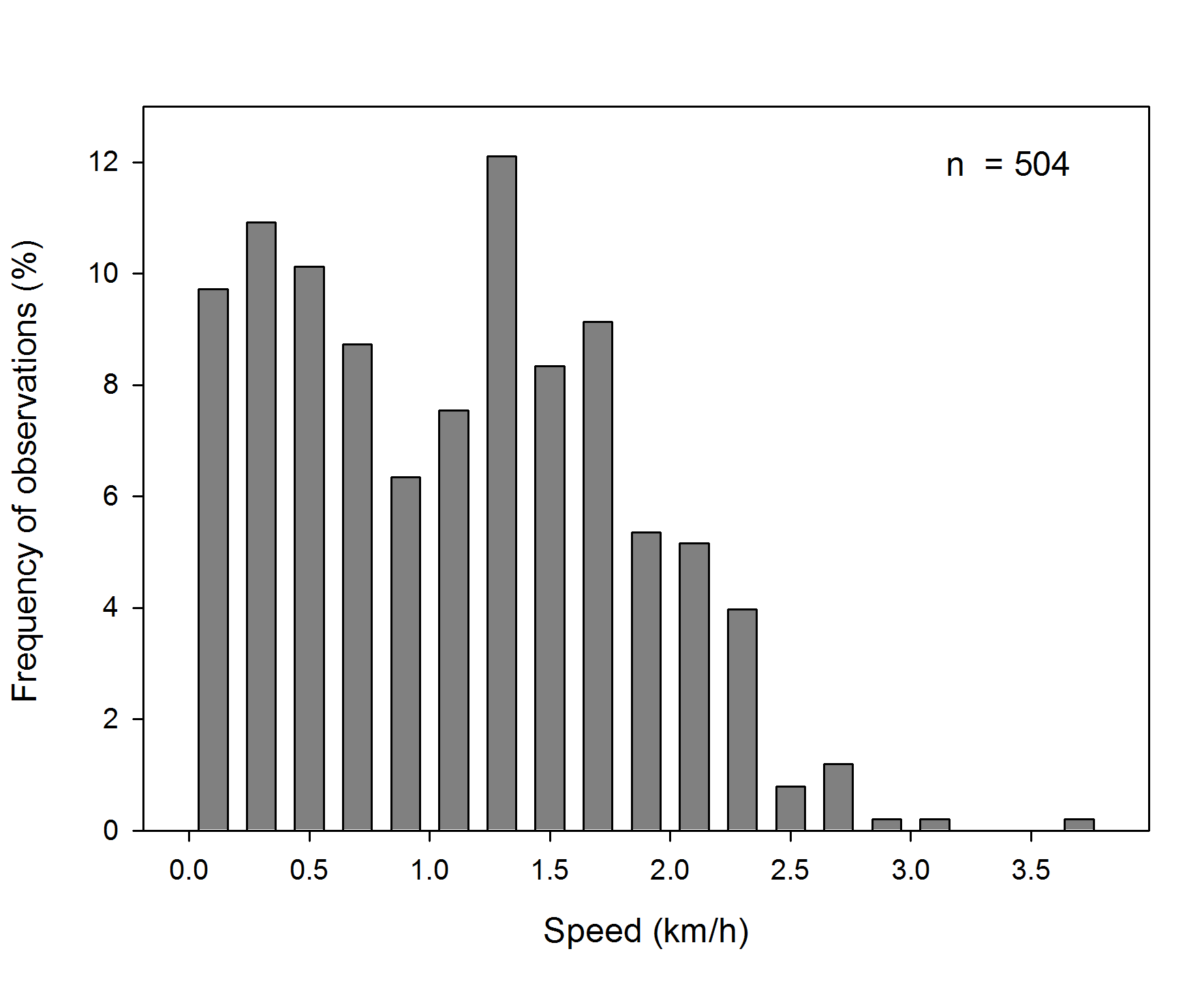

Supplement: Figure S2 — Frequency distribution of blue shark speeds (km/h). Speed (km/h) was calculated as the displacement between quality 2 and 3 Argos locations separated by time intervals of 1–4 days. (TIF) [file pone.0103538.s002.tif]
